# P162: Behavior and infection control/ influencing healthcare workers

**DOI:** 10.1186/2047-2994-2-S1-P162

**Published:** 2013-06-20

**Authors:** PH Mishra

**Affiliations:** 1 Medical Superintendent, Indian Spinal Injuries Centre, Delhi, India

## Introduction

Health care-associated infections (HAIs) have become more common 5-8% (30% is preventable) as medical care has grown more complex and patients have become more knowledgeable and demanding.

## Objectives

The objective of this study was to evaluate and suggest for improvement in Health Care Acquired infection at 150 bedded Indian Spinal Injuries Centre (ISIC).

## Methods

Method included a retrospective study of HAI data for 2 years (2011-2012).

## Results

Result showed that Catheter Associated Blood Stream Infection (CA-BSI) in 2011 was 0.2% & in 2012 1.3%. CA UTI 2011 was 3% & in 2012 was 3.5%. E.Coli 52% is main cause of Urine infections & most sensitive to Colistin, Imipenam, Kleibsella 32%, Pseudomonas 15% more sensitive to Imipenam and Tazobactum.

Respiratory infection E. Coli 33%, Staph 33%, Acinetobacter 34% CDC 4.8-6/1000 Ventilator days. More sensitive to Colistin.

VAP rates in ISIC in 2011 was 5% & in 2012 was 2.6%.

## Conclusion

### Interventions

To be effective the infections control program has included the following:

Organized surveillance and control activities.

Two infection control Nurses have been appointed.

A Trained Hospital Epidemiologist & Microbiologist have been appointed.

A system for reporting surgical wound infection rates and other infection back to the practicing surgeons and physicians developed.

### UTI – Guideline

Booklet distributed, bed side teaching is done for Catheter care, CIC Counseling, Antibiotic Catheter, VAP- Raised head end 30%, Culture 1^st^ day & 3^rd^ Day leads to early detection and treatment.

SSI-Stopped shaving, clipping done, Chlorhexidine & Betadine used for skin preparation.

## Disclosure of interest

None declared

